# Cesarean section under spinal anesthesia in acquired complete atrioventricular block without a pacemaker: A case report

**DOI:** 10.1002/ccr3.2312

**Published:** 2019-07-24

**Authors:** Shungo Aratake, Atsushi Yasuda, Shigehito Sawamura

**Affiliations:** ^1^ Department of Anesthesiology Teikyo University School of Medicine Tokyo Japan

**Keywords:** cesarean section, complete atrioventricular block, pacemaker, spinal anesthesia

## Abstract

Pregnancy with complete atrioventricular block is rare, and its perioperative management is controversial. We successfully managed cesarean section in a pregnancy with acquired complete atrioventricular block under spinal anesthesia without a pacemaker. Asymptomatic pregnant women with acquired complete atrioventricular block can tolerate cesarean section under spinal anesthesia without a pacemaker.

## 1 INTRODUCTION

Complete atrioventricular block (CAVB) is defined as disruption of electrical excitation in the atrioventricular conduction system. CAVB is generally classified as congenital or acquired; the former is associated with heart malformations or maternal autoimmune disease, and the latter is derived from cardiac surgery, rheumatic heart disease, or an infective disorder.^1^ In severe cases with clinical symptoms, pacemaker (PM) implantation becomes necessary. Pregnancy itself induces changes in hemodynamics, and pregnant women with CAVB without a PM, especially those with acquired CAVB, may experience drastic changes in hemodynamics, requiring careful management. However, there is no clear guideline for the perioperative management of acquired CAVB, including implantation of a PM and anesthesia methods. We report anesthetic management for cesarean section (C‐section) in a woman with acquired CAVB without implanting a PM.

## 2 CASE PRESENTATION

We obtained written informed consent for the present report from the patient and her family.

### 2.1 Case history and examination

**Figure 1:**
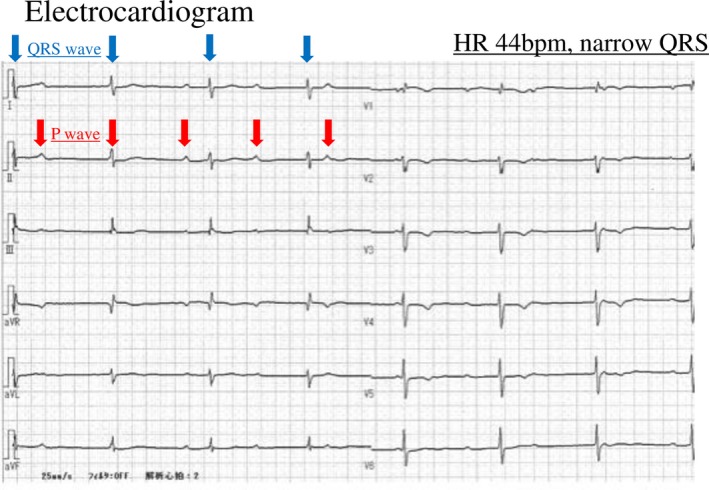
Preoperative electrocardiogram (complete atrioventricular block). The QRS did not follow the P wave. Heart rate was 44 bpm, and QRS width was 94 ms

A 23‐year‐old primigravida through natural conception was considered high risk for delivery because of atrial septal defect closure at 6 years of age complicated by CAVB. Her height and weight were 153 cm and 48 kg (increased to 57 kg during pregnancy), respectively. No previous medical history was noted, other than the cardiac surgery, and she had no history of drug addiction. A permanent PM was not implanted because she showed no symptoms and refused the procedure. Her fetus followed a normal course of development, with no signs of growth retardation. During regular antenatal checkups, she remained asymptomatic without medication, had no restriction on exercise in daily life, and received no contraceptive advice. Her blood pressure remained at about 100/55 mm Hg, and proteinuria was absent. A Holter monitor before pregnancy showed a minimum heart rate (HR) of 34 beats/minute (bpm) and a maximum HR of 82 bpm (mean, 50 bpm). Cardiotocography and echocardiography of the fetus showed no abnormalities. Because of acquired CAVB and the patient's request, an elective C‐section was chosen after discussion with multidisciplinary professionals, including a cardiologist. C‐section was scheduled at 37 weeks and 4 days of gestational age. A preoperative 12‐lead electrocardiogram showed CAVB in which not every QRS complex followed a P wave. The HR was 44 bpm, and the QRS duration was 94 ms (Figure 1). Blood test results were all within normal limits, except for atrial natriuretic peptide and brain natriuretic peptide, which showed small increases of 104.6 pg/mL and 101 pg/mL, respectively, but were unchanged compared with prepregnancy values. Transthoracic echocardiography showed an ejection fraction of 61% and no regional wall motion abnormalities. Interatrial shunt was absent, but mild mitral valve insufficiency and tricuspid valve insufficiency were noted.

We discussed prophylactic insertion of a temporary PM with the patient. She and her family were concerned about the risks and refused prophylactic insertion of a temporary PM. Therefore, surgery was planned without a temporary PM. The cardiologist stated that a temporary PM could be inserted within less than a minute, which also supported our plan.

### 2.2 Treatment

**Figure 2:**
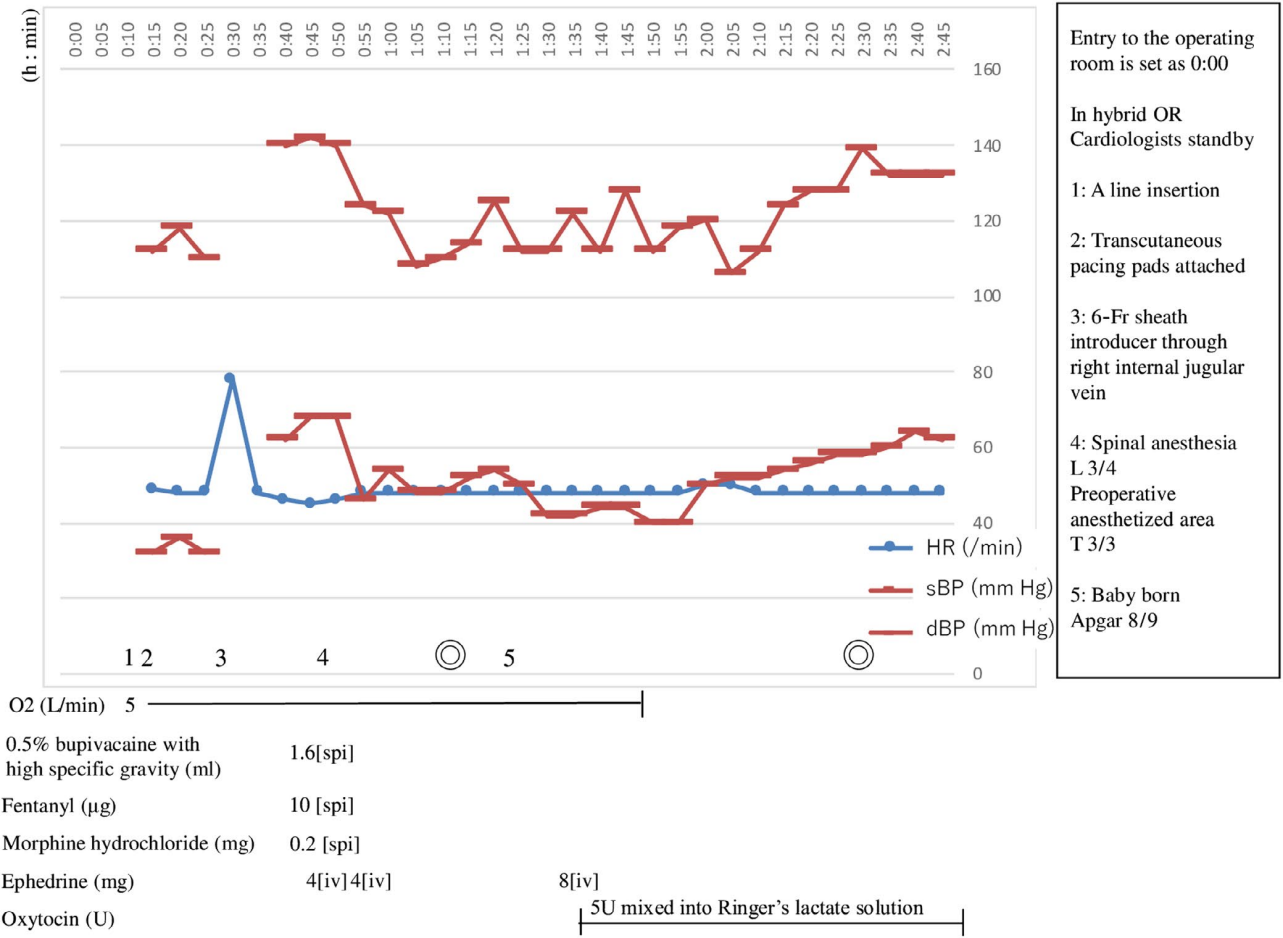
Anesthesia record. dBP, diastolic blood pressure; HR, heart rate; it, intrathecal; iv, intravenous administration; OR, operating room; sBP, systolic blood pressure

The surgical course is shown in Figure 2. The patient entered the hybrid operating room with a cardiologist standing by in the room. Transcutaneous pacing pads were attached, atropine and isoproterenol were prepared in the operating room, and then, an arterial line was inserted into the left radial artery. After local anesthesia with 1% lidocaine, a 6‐French sheath introducer was inserted through the right internal jugular vein for access if emergency transvenous pacing was needed. The patient was placed in the right lateral decubitus position. After local anesthesia with 1% lidocaine, a mixture of hyperbaric bupivacaine (8 mg/1.6 mL), fentanyl (10 μg/0.2 mL), and morphine hydrochloride (0.2 mg/0.2 mL) was injected into the subarachnoid space at the L3‐L4 level using a 25‐gauge Quincke spinal needle. She was moved back to the supine position. Ephedrine (4 mg) was administered twice, and the reaction of her HR to the drug was checked twice; her HR and hemodynamic status remained unchanged following drug administration. After loss of bilateral cold sensation up to the T3 level was confirmed, the surgery was started. C‐section was completed without major disturbances in the patient's hemodynamic status; the systolic blood pressure was 100‐140 mm Hg with an HR of approximately 50 bpm. The duration of anesthesia was 1 hour and 44 minutes, while the surgical time was 1 hour and 21 minutes. Blood loss, including amniotic fluid, was about 400 mL. Approximately 1700 mL of Ringer's lactate solution was infused. The newborn was female, weighed 2500 g (−0.4 standard deviation). Apgar scores were 8 and 9 at 1 and 5 minutes, respectively.

### 2.3 Outcome and follow‐up

The patient was transferred to the intensive care unit. Her postoperative hemodynamic status, including HR, was stable, and she was transferred to the general ward the day after surgery. She developed an intrauterine infection and required intravenous antibiotic treatment. She was discharged on postoperative day 18 after the infection resolved. The newborn had no rhythm disturbance or congenital heart disease and was discharged on the same day.

## 3 DISCUSSION

Pregnancy is characterized by an increased circulating plasma volume and cardiac output compared with the nongravid state. When pregnancy is complicated by CAVB, heart failure symptoms may worsen as pregnancy progresses; thus, careful management is necessary.^2^ Temporary PMs have been routinely inserted for those pregnancies without a PM. However, its necessity has not yet been discussed enough.^3^ Previous reports have described the indication of temporary PMs in patients undergoing C‐section with congenital CAVB. Some of them suggested that implantation of a PM prior to C‐section is unnecessary in an asymptomatic pregnant with congenital CAVB.^3^, ^4^, ^5^, ^6^ However, case reports describing C‐section in pregnancy with acquired CAVB without insertion of a temporary PM are limited.^4^, ^7^ This is probably because the prognosis in acquired CAVB is generally worse than that in congenital CAVB.^3^ Ventricular escape beats arise from more distal parts of the conduction system in acquired CAVB than in congenital CAVB, and the QRS complex tends to be wider, which makes the HR response poor to medical agents and sympathetic nerve stimulation,^8^ and makes cardiac contraction dyssynchronous between the left and right sides of the heart, leading to low cardiac output. In the present case, the QRS complex was narrow, suggesting that the origin of the escape rhythm was proximal to the bundle branches. Thus, we considered that this C‐section could be managed without a temporary PM prophylactically.

As for anesthetic management in pregnancy with acquired CAVB, general anesthesia or epidural anesthesia have been chosen in the reported cases^4^, ^7^; however, we found no prior report of spinal anesthesia alone in acquired CAVB pregnancies. Indeed, rapid onset of spinal anesthesia could cause hemodynamic instability. Furthermore, blocking of a cardiac branch of the sympathetic trunk could induce bradycardia and the Bezold‐Jarisch reflex could exacerbate bradycardia and hypotension.^6^, ^7^, ^8^ Together, these factors could explain avoidance of spinal anesthesia. However, it may not be suitable to choose epidural anesthesia in an emergent situation because it takes much more time than spinal anesthesia. General anesthesia with inhaled or intravenous agents could have potential risks of altering the hemodynamic status significantly.^6^ Therefore, it is necessary to consider the indication of spinal anesthesia in pregnancies with acquired CAVB. Kumar et al reported successful management of C‐sections in asymptomatic congenital CAVB under spinal anesthesia with PM placement,^5^ and Mohapatra et al reported successful management of emergent C‐section in asymptomatic congenital CAVB using spinal anesthesia without a PM.^3^ Sundararaman et al also reported successful management of vacuum‐assisted delivery in asymptomatic congenital CAVB under combined spinal‐epidural anesthesia.^6^ Thus, management for C‐sections in acquired CAVB should not be limited to general or epidural anesthesia. In our case, the patient did not have exercise restriction before pregnancy. Preoperative test results and her hemodynamic status remained stable even after pregnancy. Thus, we considered that the patient could tolerate C‐section under spinal anesthesia and decided not to place a temporary transvenous PM prophylactically.

Hemodynamic instability during C‐section is unpredictable^8^; thus, surgeons and anesthesiologists should prepare for emergent hemodynamic instability. Sundararaman et al reported an example of preparation for hemodynamic instability in patients with asymptomatic congenital CAVB.^6^ For example, in our case, we placed transcutaneous pacing pads and a transvenous PM access route and kept a cardiologist on standby in the hybrid operating room so that we could manage the patient's hemodynamics safely in the event of severe bradycardia.

Because of lack of consensus, the anesthesiologist should evaluate the risk of fluctuations in hemodynamics before surgery through consultation with the obstetrician and cardiologist, and should determine the indication for a temporary PM and anesthetic methods on a case‐by‐case basis. Case accumulation is essential in order to consider the indication for PMs and appropriate anesthetic management in patients with acquired CAVB without permanent PM.

## CONFLICT OF INTEREST

None.

## AUTHOR CONTRIBUTIONS

SA: wrote the manuscript. AY: helped with the literature review for the case preparation and manuscript preparation. SS: helped with the literature review for the case preparation and manuscript preparation. All authors read and approved the final manuscript.
